# Telomere Shortening Unrelated to Smoking, Body Weight, Physical Activity, and Alcohol Intake: 4,576 General Population Individuals with Repeat Measurements 10 Years Apart

**DOI:** 10.1371/journal.pgen.1004191

**Published:** 2014-03-13

**Authors:** Maren Weischer, Stig E. Bojesen, Børge G. Nordestgaard

**Affiliations:** 1 Department of Clinical Biochemistry, Herlev Hospital, Copenhagen University Hospital, Copenhagen, Denmark; 2 Faculty of Health and Medical Sciences, University of Copenhagen, Copenhagen, Denmark; 3 The Copenhagen City Heart Study, Frederiksberg Hospital, Copenhagen University Hospital, Copenhagen, Denmark; Georgia Institute of Technology, United States of America

## Abstract

Cross-sectional studies have associated short telomere length with smoking, body weight, physical activity, and possibly alcohol intake; however, whether these associations are due to confounding is unknown. We tested these hypotheses in 4,576 individuals from the general population cross-sectionally, and with repeat measurement of relative telomere length 10 years apart. We also tested whether change in telomere length is associated with mortality and morbidity in the general population. Relative telomere length was measured with quantitative polymerase chain reaction. Cross-sectionally at the first examination, short telomere length was associated with increased age (*P* for trend across quartiles = 3×10^−77^), current smoking (*P* = 8×10^−3^), increased body mass index (*P* = 7×10^−14^), physical inactivity (*P* = 4×10^−17^), but not with increased alcohol intake (*P* = 0.10). At the second examination 10 years later, 56% of participants had lost and 44% gained telomere length with a mean loss of 193 basepairs. Change in leukocyte telomere length during 10 years was associated inversely with baseline telomere length (*P*<1×10^−300^) and age at baseline (*P* = 1×10^−27^), but not with baseline or 10-year inter-observational tobacco consumption, body weight, physical activity, or alcohol intake. Prospectively during a further 10 years follow-up after the second examination, quartiles of telomere length change did not associate with risk of all-cause mortality, cancer, chronic obstructive pulmonary disease, diabetes mellitus, ischemic cerebrovascular disease, or ischemic heart disease. In conclusion, smoking, increased body weight, and physical inactivity were associated with short telomere length cross-sectionally, but not with telomere length change during 10 years observation, and alcohol intake was associated with neither. Also, change in telomere length did not associate prospectively with mortality or morbidity in the general population.

## Author Summary

Human chromosomes are capped by protective ends called telomeres. These ends are shortened during renewal of tissue and eventually become critically short, causing cells to become senescent or die. It is widely believed that lifestyle features such as smoking, obesity, physical inactivity, and possibly alcohol intake enhance shortening of telomeres. However, strong evidence to support such an interpretation is hard to find. We therefore tested whether these lifestyle factors are associated with telomere length change in 4,576 healthy individuals from the general population. Individuals had relative telomere length measured twice with a 10-year interval, and were then followed for mortality and morbidity for a further 10 years after the second measurement. We found change in telomere length to be more dynamic than previously believed, as we observed both shortening (in 56%) and lengthening (in 44%) among participants. Contrary to previous beliefs, we found telomere length change to be unaffected by lifestyle factors. Instead, we found the strongest association between past telomere length and age with change in telomere length over 10 years. Also, we found no association between change in telomere length and risk of all-cause mortality, cancer, chronic obstructive lung disease, diabetes mellitus, ischemic cerebrovascular disease, or ischemic heart disease.

## Introduction

Telomeres are 1,500 to 15,000 basepairs long tandem repeat DNA sequences (TTAGGG)_x_ which cap at the ends of linear chromosomes [1], [2]. During mitosis, telomere length is shortened due to the inability of the DNA polymerase to complete duplication of the lacking strand [3]. Eventually, telomeres become critical short causing cells to become senescent or die [1], [3].

Several cross-sectional and prospective studies have associated short telomere length with increased risk of any early death [2], [4], [5], cardiovascular disease [2], [6], cancer [7], [8], and early death after a cancer diagnosis [9]. At the same time cross-sectional studies have reported short telomere length to be associated with lifestyle factors such as smoking, obesity, physical inactivity, and possible alcohol intake [2], [10]–[12]. These observations have led to a general belief that telomere length is shortened by these factors, and that telomere length possibly could be a marker of biological age of tissues [12]–[14]. The alleged associations are quoted repeatedly, but strong evidence to support these hypotheses is difficult to find. Therefore, the association between lifestyle factors and short telomere length could simply be due to confounding. Moreover, it is uncertain whether change in lifestyle can change telomere length, and whether telomere length change is associated with risk of mortality and morbidity.

We tested the hypothesis that smoking, body weight, physical activity, and alcohol intake are associated with telomere length cross-sectionally and with telomere length change during 10 years observation in the general population. Furthermore, we tested whether telomere length change is associated with mortality and morbidity.

## Results

### Cross-sectional: Telomere length

**Figure 1:**
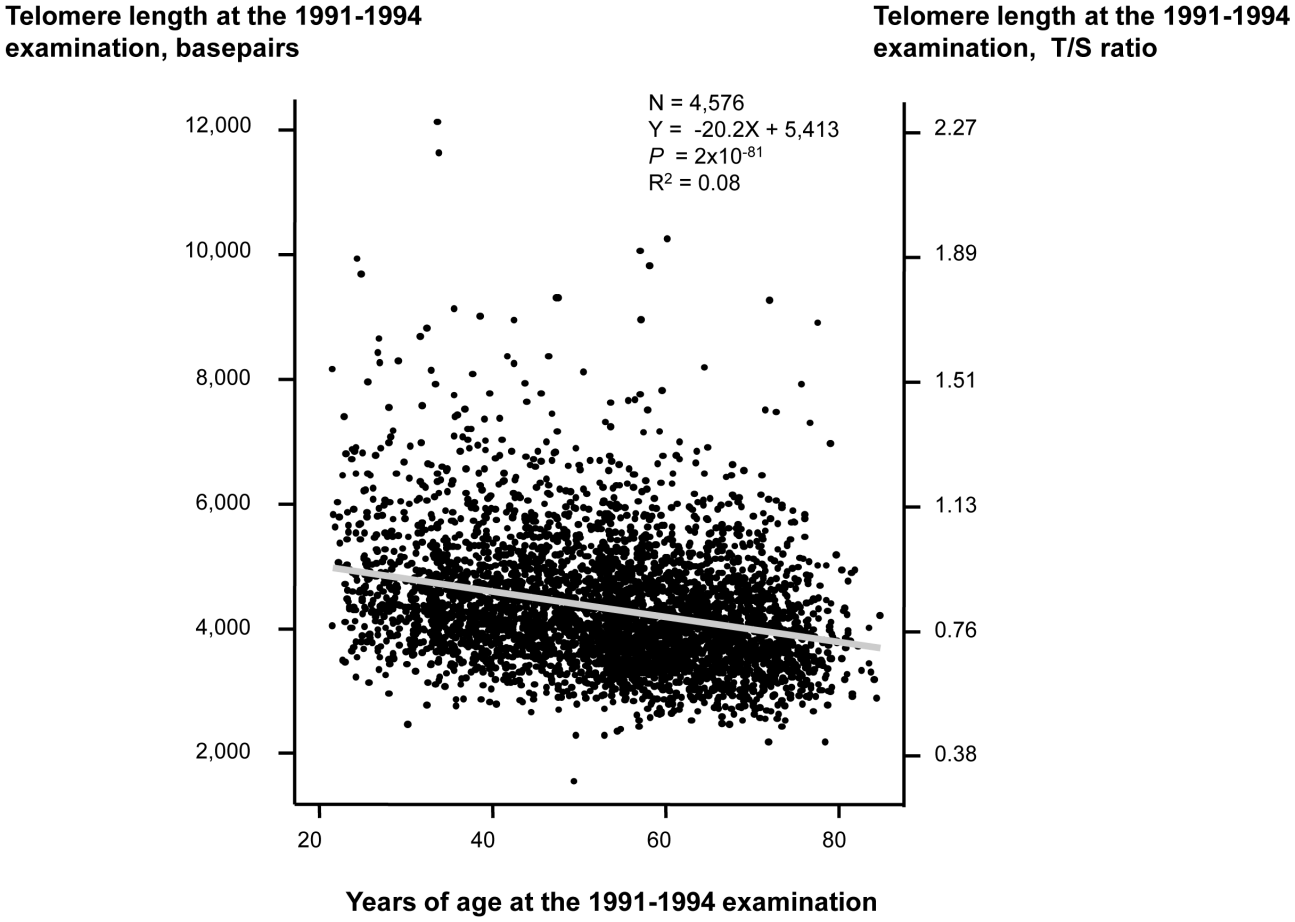
Telomere length in basepairs as a function of age in years at the 1991–1994 examination. Linear regression is shown in equation and as a grey line. N = number of participants. P-value and R^2^ are for the correlation from the linear regression. Statistical tests were two-sided.

Characteristics of participants as a function of examination specific telomere length quartiles are shown in Table 1. Cross-sectionally at the 1991–1994 examination, short telomere length was associated with increased age (*P* for trend across quartiles = 3×10^−77^), male gender (*P* = 0.02), current smoking (*P* = 8×10^−3^), increased body mass index (*P* = 7×10^−14^), physical inactivity (*P* = 4×10^−17^), but not with increased alcohol intake (*P* = 0.10). Correspondingly at the 2001–2003 examination, short telomere length was associated with increased age (*P* for trend across quartiles = 2×10^−74^), male gender (*P* = 1×10^−5^), increased body mass index (*P* = 4×10^−4^), and physical inactivity (*P* = 2×10^−33^), but not with current smoking (*P* = 0.32) or with increased alcohol intake (*P* = 0.01 for reduced intake). At study entry, we observed a 20 basepair decrease in telomere length per year of age (*P*<2×10^−81^; Figure 1). Variation in age explained 8% of the variation in telomere length (R^2^ = 0.08).

**Table 1:** Characteristics of participants in the general population, the Copenhagen City Heart Study.

	Telomere length				
	1^st^ quartile	2^nd^ quartile	3^rd^ quartile	4^th^ quartile	*P* for trend
1991–1994 examination					
Telomere lengthRelative telomere length, basepairs	12,132 to 4,806	4,804 to 4,170	4,167 to 3,670	3,668 to 1,544	
No. of participants	1,144	1,143	1,138	1,151	
Age, years	49 (38 to 59)	53 (40 to 63)	55 (45 to 65)	60 (53 to 68)	3×10^−77^
Men, n (%)	471 (41)	474 (41)	464 (41)	534 (46)	0.02
Current smoking, n (%)	461 (40)	485 (42)	495 (44)	536 (47)	8×10^−3^
Daily tobacco consumption, g *	16 (15 to 17)	15 (14 to 16)	16 (15 to 17)	16 (15 to 17)	0.32
Body mass index, kg/m^2^	24 (22 to 27)	24 (22 to 27)	25 (23 to 27)	25 (23 to 28)	7×10^−14^
Physical inactivity, n (%)**	133 (12)	163 (14)	208 (18)	202 (23)	4×10^−17^
Heavy alcohol intake, n (%)***	531 (46)	487 (43)	481 (42)	498 (43)	0.10
2001–2003 examination					
Telomere length, basepairs	12,088 to 4,714	4,713 to 4,065	4,064 to 3,461	3,460 to 1,419	
No. of participants	1,144	1,144	1,144	1,144	
Age, years	57 (47 to 67)	63 (51 to 72)	66 (56 to 75)	68 (60 to 76)	2×10^−74^
Men, n (%)	448 (39)	473 (41)	499 (44)	523 (46)	1×10^−5^
Current smoking, n (%)	368 (32)	359 (31)	394 (34)	378 (33)	0.32
Daily tobacco consumption, g *	15 (15 to 16)	16 (15 to 17)	16 (15 to 17)	15 (15 to 16)	0.12
Body mass index, kg/m^2^	25 (23 to 28)	26 (23 to 28)	26 (23 to 29)	26 (23 to 29)	4×10^−4^
Physical inactivity, n (%)**	247 (22)	348 (30)	422 (37)	499 (44)	2×10^−33^
Heavy alcohol intake, n (%)***	551 (48)	516 (45)	502 (44)	500 (44)	0.01

### 10 years observation: Telomere length change

**Figure 2:**
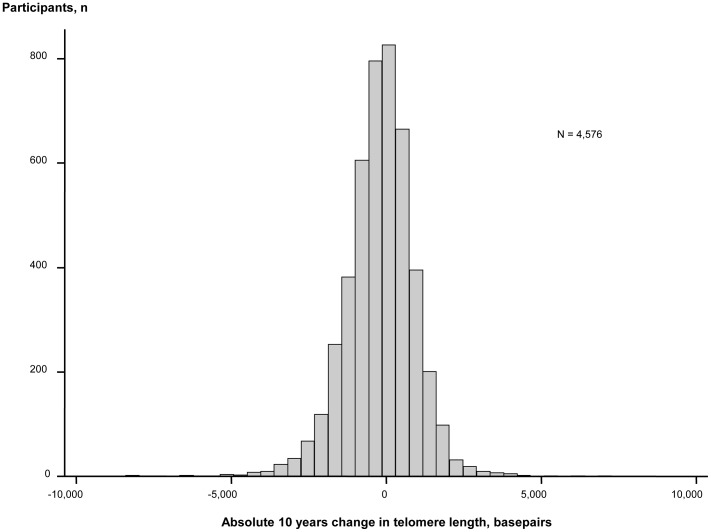
Absolute change in basepairs during 10 years observation. N = number of participants.

During 10 years observation, 56% of participants lost and 44% gained telomere length (Figure 2). Mean change in telomere length was −19.3 basepairs per year. Ten year maximum loss and gain were −8,406 and +7,278 basepairs. Characteristics of participants stratified for 10 year loss or gain of telomere length were similar (Table S1). The telomere length distribution at each examination is shown in Figure S1.

**Figure 3:**
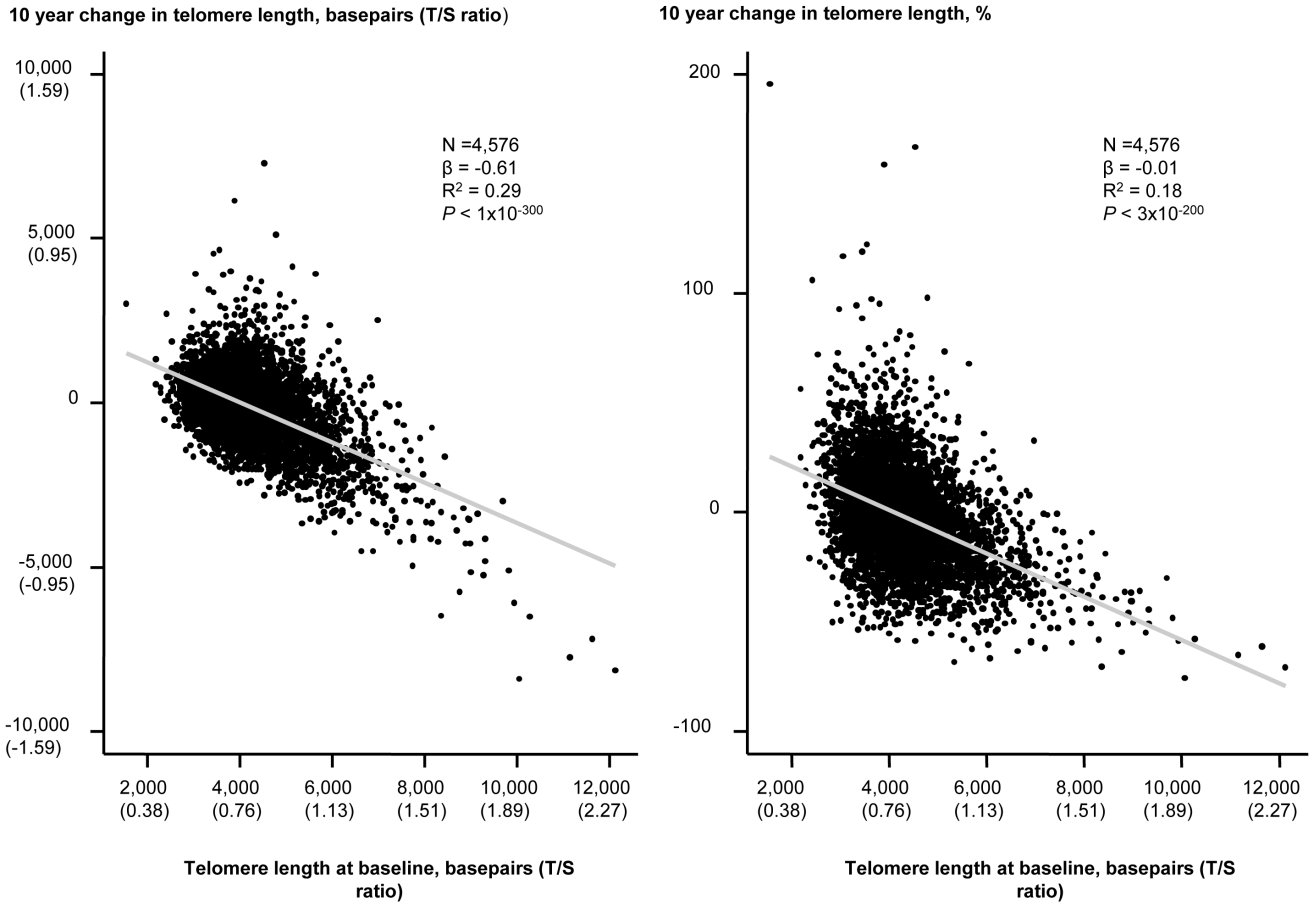
10 year change in telomere length in basepairs or percent as functions of telomere length at the 1991–1994 examination. Linear regressions are shown in equations and as grey lines. N = number of participants. P-value and R^2^ are for the correlation from the linear regression. Statistical tests were two-sided.

An inverse association of change in telomere length in either basepairs or in percent with baseline telomere length in basepairs are illustrated in Figure 3. Baseline telomere length was associated inversely with 10 years change in basepairs of telomere length, and variation in baseline telomere length explained 29% of variation in 10 years change in basepairs in telomere length (P<1×10^−300^, R^2^ = 0.29; Figure 3, left). Baseline telomere length was also associated inversely with 10 years percent change in telomere length and variation in baseline telomere length explained 18% of variation in 10 years percent change in telomere length (P = 3×10^−200^, R^2^ = 0.18; Figure 3, right).

The independent predictors of change in leukocyte telomere length over 10 years were in multivariable models baseline telomere length and age at baseline (Table 2). Stratification on 10 year telomere length gain or loss showed similar results (Table S2). In overall analysis and in sub-analyses, baseline and 10 year inter-observational tobacco consumption, body weight, physical inactivity, and alcohol intake were not associated with change in telomere length.

**Table 2:** Independent predictors of change in telomere length from the 1991–1994 to the 2001–20013 examinations.

		10 year change in telomere length, basepairs			10 year change in telomere length, %		
	Participants, n	β-coefficient	95% confidence interval	*P*-value	β-coefficient	95% confidence interval	*P*-value
**Univariable analysis**							
Baseline telomere length, basepairs or %	4,576	−0.61	−0.64 to −0.58	<1×10^−300^	−0.01	−0.01 to −0.01	3×10^−200^
Baseline T/S ratio	4,576	−3,226	−3,373 to −3,078	<1×10^−300^	−52	−55 to −49	3×10^−200^
Age at baseline, years	4,576	−0.29	−2.69 to 2.12	0.81	−0.03	−0.07 to 0.02	0.32
Baseline daily tobacco consumption, g/day	4,576	4.05	−0.97 to 7.13	0.01	0.05	−0.02 to 0.12	0.15
Tobacco consumption between examinations, g/day	4,576	2.83	−0.97 to 6.63	0.15	0.06	−0.02 to 0.13	0.15
Baseline weight, kg	4,569	0.89	−1.50 to 3.27	0.47	0.02	−0.03 to 0.06	0.52
Weight change, %	4,567	1.30	−2.48 to 5.09	0.67	0.04	−0.04 to 0.12	0.31
Baseline alcohol intake, units*/day	4,535	−0.84	−3.89 to 2.21	0.59	−0.01	−0.07 to 0.05	0.81
Alcohol intake between examinations, units*/day	4,381	−1.27	−4.69 to 2.15	0.47	−0.01	−0.08 to 0.06	0.70
Baseline physicial activity, hours/week	4,035	−8.49	−33.37 to 16.39	0.50	−0.09	−0.60 to 0.42	0.74
Physical activity between examination, hours/week	4,035	−2.71	−31.39 to 25.97	0.85	0.12	−0.47 to 0.70	0.70
**Multivariable analysis**							
Baseline telomere length, basepairs or %	4,576	−0.66	−0.69 to −0.63	<1×10^−300^	−0.01	−0.01 to −0.01	5×10^−195^
Baseline T/S ratio	4,576	−3,512	−3,667 to −3,388	<1×10^−300^	−53	−57 to −50	5×10^−195^
Age at baseline, years	4,576	−14.04	−16.55 to −11.53	1×10^−27^	−0.25	−0.31 to −0.20	4×10^−19^
Baseline daily tobacco consumption, g/day	4,576	−2.91	−11.35 to 5.57	0.50	−0.01	−0.20 to 0.17	0.88
Tobacco consumption between examinations, g/day	4,576	3.44	−5.95 to 12.83	0.41	0.03	−0.17 to 0.24	0.74
Baseline weight, kg	4,569	−1.04	−3.12 to 1.03	0.32	−0.02	−0.07 to 0.03	0.55
Weight change, %	4,567	1.59	−1.91 to 5.09	0.38	−0.01	−0.06 to 0.03	0.40
Baseline alcohol intake, units*/day	4,535	−1.54	−7.38 to 4.30	0.61	−0.02	−0.15 to 0.11	0.84
Alcohol intake between examinations, units*/day	4,381	−1.12	−7.50 to 5.26	0.73	−0.03	−0.17 to 0.11	0.72
Baseline physicial activity, hours/week	4,035	−12.79	−35.02 to 9.44	0.26	−0.15	−0.64 to 0.34	0.57
Physical activity between examination, hours/week	4,035	−1.92	−32.30 to 28.45	0.90	−0.19	−0.86 to 0.47	0.57

* One unit alcohol equals 12 g. The rate of change in leukocyte telomere length is negative; therefore, negative regression coefficients denote faster telomere length loss. Baseline values were obtained at the 1991–94 examination.

The fact that telomere length at baseline was the only factor strongly associated with change in telomere length prompted us to investigate whether the telomere length change could be partly explained by regression towards the mean, ie. that the combined effect of biological and analytical variability will push the highest levels to a lower level on retesting and *vice versa* for those with the lowest levels [15]. This was indeed the case with a regression dilution ratio of 0.50 for the quartilation used in this study (Figure S2).

### Prospective studies: Mortality and morbidity

Participants were grouped in quartiles according to change in telomere length over two examinations spanning 10 years, and were then followed for an additional up to 10 years after the second examination (Table 3). Median follow-up after the second examination were 8.1 years for survival, 6.7 years for cancer, and 7.9 years for chronic obstructive pulmonary disease, diabetes mellitus type II, ischemic cerebrovascular disease, and ischemic heart disease. We observed no association between quartiles of telomere length change and risk of early death (*P* for trend across quartiles = 0.21), cancer (*P* = 0.62), chronic obstructive pulmonary disease (*P* = 0.60), diabetes mellitus type II (*P* = 0.37), ischemic cerebrovascular disease (*P* = 0.60), or ischemic heart disease (*P* = 0.56). Stratification on 10-year telomere length gain or loss showed similar results (Table S3).

**Table 3:** Mortality and morbidity by quartiles of telomere length change.

	10 year change in telomere length				Hazard ratio (95% confidence inteval)		
	Basepairs	T/S ratio	Participants, n	Events, n	Unadjusted	Multivariably adjusted	*P* for trend
All cause mortality							
1^st^ quartile	479 to 7,278	0.09 to 1.38	1,144	186	1.00	1.00	0.21
2^nd^ quartile	−137 to 478	−0.02 to 0.08	1,144	240	1.17 (0.97 to 1.42)	1.13 (0.93 to 1.37)	
3^rd^ quartile	−801 to −136	−0.14 to −0.03	1,144	223	1.10 (0.91 to 1.34)	1.02 (0.84 to 1.24)	
4^th^ quartile	−8,406 to −800	−1.59 to −0.15	1,144	227	1.17 (0.97 to 1.42)	1.18 (0.97 to 1.44)	
Cancer							
1^st^ quartile	479 to 7,278	0.09 to 1.38	1,116	152	1.00	1.00	0.62
2^nd^ quartile	−137 to 478	−0.02 to 0.08	1,118	166	1.10 (0.84 to 1.43)	1.09 (0.83 to 1.42)	
3^rd^ quartile	−801 to −136	−0.14 to −0.03	1,103	137	0.81 (0.61 to 1.08)	0.77 (0.58 to 1.04)	
4^th^ quartile	−8,406 to −800	−1.59 to −0.15	1,111	179	1.18 (0.90 to 1.54)	1.19 (0.91 to 1.55)	
Chronic obstructive pulmonary disease							
1^st^ quartile	479 to 7,278	0.09 to 1.38	1,139	90	1.00	1.00	0.60
2^nd^ quartile	−137 to 478	−0.02 to 0.08	1,140	111	1.28 (0.92 to 1.77)	1.22 (0.88 to 1.70)	
3^rd^ quartile	−801 to −136	−0.14 to −0.03	1,142	102	1.15 (0.82 to 1.60)	1.03 (0.74 to 1.44)	
4^th^ quartile	−8,406 to −800	−1.59 to −0.15	1,137	108	1.09 (0.78 to 1.54)	1.17 (0.83 to 1.65)	
Diabetes Mellitus type II							
1^st^ quartile	479 to 7,278	0.09 to 1.38	1,134	66	1.00	1.00	0.37
2^nd^ quartile	−137 to 478	−0.02 to 0.08	1,137	76	1.19 (0.79 to 1.80)	1.13 (0.75 to 1.71)	
3^rd^ quartile	−801 to −136	−0.14 to −0.03	1,135	81	1.21 (0.81 to 1.82)	1.16 (0.77 to 1.75)	
4^th^ quartile	−8,406 to −800	−1.59 to −0.15	1,134	79	1.18 (0.79 to 1.79)	1.21 (0.80 to 1.83)	
Ischemic cerebrovascular disease							
1^st^ quartile	479 to 7,278	0.09 to 1.38	1,134	68	1.00	1.00	0.60
2^nd^ quartile	−137 to 478	−0.02 to 0.08	1,131	79	1.07 (0.73 to 1.55)	1.06 (0.73 to 1.55)	
3^rd^ quartile	−801 to −136	−0.14 to −0.03	1,134	78	0.93 (0.63 to 1.37)	0.90 (0.61 to 1.33)	
4^th^ quartile	−8,406 to −800	−1.59 to −0.15	1,133	70	0.95 (0.64 to 1.41)	0.95 (0.64 to 1.40)	
Ischemic heart disease							
1^st^ quartile	479 to 7,278	0.09 to 1.38	1,123	164	1.00	1.00	0.56
2^nd^ quartile	−137 to 478	−0.02 to 0.08	1,109	163	0.91 (0.69 to 1.19)	0.89 (0.68 to 1.17)	
3^rd^ quartile	−801 to −136	−0.14 to −0.03	1,122	177	1.02 (0.78 to 1.33)	0.98 (0.75 to 1.27)	
4^th^ quartile	−8,406 to −800	−1.59 to −0.15	1,118	184	1.04 (0.80 to 1.35)	1.06 (0.81 to 1.38)	

Multivariable adjustment included covariates obtained at the 2001–2003 examination; age, gender, current smoking, daily tobacco consumption, body mass index, heavy alcohol intake, and physical inactivity.

**Figure 4:**
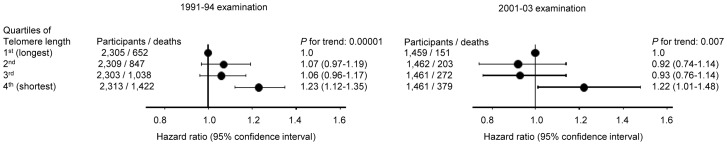
All-cause mortality by quartiles of telomere length at the 1991–94 examination (left) and at the 2001–03 examination (right). Hazard ratios were adjusted for covariates obtained at the date of examination; age, gender, current smoking, daily tobacco consumption, body mass index, heavy alcohol intake, and physical inactivity. Numbers of participants were all available participants with measured telomere lengths (1991–94; 9250, 2001–03; 5843). Follow-up began the day of the 1991–94 (for the 9250 participants examined in 1991–94) or the 2001–03 (for the 5843 participants examined in 2001–03) examination.

In contrast, short telomere length was associated with all-**cause** mortality both at the 1991–94 examination (*P* = 0.00001) and at the 2001–03 examination (*P* = 0.007) (Figure 4), illustrating that the telomere length measurement included biological important information at both examinations.

## Discussion

We found association of smoking, increased body weight, and physical inactivity with short telomere length cross-sectionally, but not with telomere length change during 10 years observation, while alcohol intake was associated with neither. Also, change in telomere length did not associate prospectively with mortality or morbidity in the general population, whereas both cross-sectional measurements did associate with mortality.

It it is somewhat surprising that we observed telomere elongation in nearly half of our participants. This suggests that telomere length is more dynamic than hitherto acknowledged, and that non-stem cells may have an underestimated regenerative potential. Although, telomere elongation is not widely discussed, other prospective studies have reported telomere elongation in 11% to 33% of adult individuals [16]–[19], not so different from the 44% with telomere elongation in the present study as we did not consider a middle group with unchanged telomere length. Interestingly, tropical python hatchlings are born with short telomere length and extend their telomeres as they grow. A slight gender bias, similar to what is observed cross-sectionally in humans, causes longer telomere in adult female pythons [20]. The observed telomere elongation may occur through telomerase activity or through other pathways. In support of the telomerase pathway, telomerase activity has been reported in some studies of non-malignant peripheral mononuclear blood cells [21], but not in all studies [22]–[24]. The last notion is supported by the observations that in tumors, where telomerase activity is present as opposed to their normal tissue counterparts, the telomeres are shorter [22]–[24]. On the other hand, we cannot completely exclude that our observed telomere elongation to some extent could be an artifact, either caused by a redistribution of leukocyte subpopulations between tissues, as telomere length vary among lymphocyte subpopulations [23], [25] or other factors [26]. But as more studies using different assays report telomere elongation [16]–[19], the possibility of telomere elongation being an artifact seems to diminish. Nevertheless, it is possible that telomere elongation in some individuals simply is explained by regression toward the mean, as seen for most measurements of biological parameters [15]. Biological and analytical variability combined will cause individuals with the highest levels to have a lower level on retesting and *vice versa* for those with the lowest levels (the latter will lead to apparent telomere elongation, when in fact it could simply represent regression toward the mean). In support of this possibility, telomere length at baseline was the only factor strongly associated with change in telomere length: according to regression toward the mean, those with the longest telomeres on the first examination will get the largest decrease in telomere length on retesting 10 years later, while those with the shortest telomeres on the first examination will get the largest increase in telomere length on retesting 10 years later. This was indeed the case, suggesting that future prospective studies of telomere length and risk of disease should probably be corrected for regression dilution bias as is common practice for many risk factor studies [15].

Most surprisingly, we were able to confirm strong associations between lifestyle and short telomere length cross-sectionally, but prospectively lifestyle did not associate with telomere length change. How can this be, when numerous reports have associated lifestyle and telomere length cross-sectionally, even the present study? We speculate that a part of the answer could be collider bias [27], a special kind of selection bias. In our study, all-cause mortality and morbidity is a collider since these outcomes are associated with short telomeres, and at the same time might reduce the likelihood for the participant of being (re)examined. Therefore, individuals with short telomeres were probably underrepresented already at the 1991–94 as well as at the 2001–03 examination, and the participants with short telomeres could theoretically be strongly selected survivors despite their short telomeres. Nevertheless, as short telomeres were associated with all-cause mortality after both examinations, this potential bias was not strong enough to eliminate the association between short telomeres and increased all-cause mortality. Therefore, we still consider that the lack of association between telomere length change and lifestyle to be plausible.

Prospectively, we found no association between change in telomere length and mortality and morbidity. This is in contrast to cross-sectional and prospective studies reporting increased risk of early death in this and other studies [4], [5], early death after a cancer diagnosis [9], cardiovascular disease [2], [6], chronic obstructive pulmonary disease [28], [29], diabetes mellitus type II [2], ischemic cerebrovascular disease [30], [31], and ischemic heart disease [2], [6] in individuals with the shortest telomeres at baseline.

Strengths of the present study include study size as we examined 4,576 individuals from the general population, and follow-up time as individuals were observed on average for 9.3 years for telomere length change and then followed for up to 10 years more for mortality and morbidity. Next, telomere length was measured with a very precise assay and the measurements were validated through inverse association with age, while the measurement of change in telomere length over 10 years was validated through association with baseline telomere length, as reported previously [2], [9]. And finally, due to the national Danish Civil Registration System, the national Danish Causes of Death Registry, and the national Danish Patient Registry, we had complete information on mortality and morbidity without losses to follow-up of even a single individual.

Limitations of the present study include that we examined whites only, and therefore our results may not necessarily be applicable to other ethnicities; however, we are not aware of data to suggest that our results should not be applicable to all races. Also, we measured telomere length in leukocytes from peripheral blood only, and not in all cell types in the body; however, leukocyte telomere length correlate highly with that in cells from other tissues [10], [32]–[34]. Finally, it could be argued that qPCR measures telomere content rather than telomere length, due to the nature of the method.

In conclusion, smoking, body weight, and physical inactivity were associated with short telomere length cross-sectionally, but not with telomere length change during 10 years observation, while alcohol intake was associated with neither. Also, change in telomere length did not associate prospectively with mortality or morbidity in the general population.

## Methods and Materials

### Ethics statement

The study was approved by Herlev Hospital and by Danish ethical committees (No. KF-V.100.2039/91, KF-01-144/01), and were conducted according to the Declaration of Helsinki. Participants gave written informed consent.

### Study participants

Participants were individuals from the prospective Copenhagen City Heart Study. All participants were white and of Danish descent. They were randomly selected from the national Danish Civil Registration System to reflect the adult Danish population ranging from age 20 to 100 years. The 4,576 participants in the present study participated both in the 1991–94 and 2001–03 examinations, and gave blood samples for DNA extraction at both examinations. Participation rate for the Copenhagen City Heart Study was 55%.

### Covariates

Prior to examinations, participants filled in self-administered questionnaires concerning present and past life-style and health status. This was completed together with examiners at the day of examination, followed by physical examination and blood sampling. The following covariates were obtained at both examinations: age in years, gender, current smoking (yes/no), daily tobacco consumption (g/day), body mass index (measured weight in kilograms divided by squared measured height in meters), heavy alcohol intake (no/yes) (yes if female and male participants reported a weekly alcohol intake above 87.5 g and 175 g), and physical inactivity (no/yes) (yes if participants reported less than 4 hours weekly exercise).

### Ascertainment of endpoints

Information on vital status from day of the second blood sampling at the 2001–03 examination until June 7^th^ 2011 was obtained from the national Danish Civil Registration System. Diagnosis of invasive cancer from January 1^st^ 1943 through December 31^st^ 2009 were obtained from the Danish Cancer Registry, which identifies 98% of all cancers diagnosed in Denmark [35], [36]. Diagnoses were classified according to the World Health Organization's International Classification of Diseases (ICD). Diagnoses of cancer were ICD-10 codes C00–D09. Diagnoses of chronic obstructive pulmonary disease, diabetes mellitus type II, ischemic cerebrovascular disease, and ischemic heart disease were obtained from January 1^st^ 1977 through May 10^th^ 2011 from the national Danish Patient Registry and the national Danish Causes of Death Registry, public registers to which all hospitalizations and deaths in Denmark are reported. Diagnoses of chronic obstructive pulmonary disease were ICD-8 codes 491–492 and ICD-10 codes J41–J44. Diagnoses of diabetes mellitus type II were ICD-8 codes 249–250 and ICD10 codes E10–E14. Diagnoses of ischemic cerebrovascular disease were ICD-8 codes 431–438 and ICD-10 codes I61-I66 and G45. Diagnoses of ischemic heart disease were ICD-8 codes 410–414 and ICD-10 codes I20-I25. For all registries follow-up was 100% complete, that is, we did not lose track of even a single individual.

### Relative telomere length

We measured relative telomere length in DNA extracted from leukocytes in peripheral blood. Relative telomere length was measured on a CFX384 real-time PCR detection system (Bio-Rad Laboratories, Denmark), using a modified Monochrome Multiplex Quantitative PCR method [37]. In the duplex PCR, the single-copy gene *ALB* which encodes albumin was amplified simultaneously with the telomere template in the same well and was used as a reference to adjust for different amounts of DNA in different samples. Final concentrations of PCR reagents were 1X QuantiFast SYBR Green Master Mix (Qiagen), 20 ng DNA, 300 nmol/L forward telomere primer (5′-ACACTAAGGTTTGGGTTTGGGTTTGGGTTTGGGTTAGTGT-′3), 300 nmol/L reverse telomere primer (5′-TGTTAGGTATCCCTATCCCTATCCCTATCCCTATCCCTAACA-′3), 350 nmol/L forward albumin primer (5′-CGGCGGCGGGCGGCGCGGGCTGGGCGGAAATGCTGCACAGAATCCTTG-′3), and 350 nmol/L reverse albumin primer (5′-GCCCGGCCCGCCGCGCCCGTCCCGCCGGAAAAGCATGGTCGCCTGTT-′3) in a volume of 10 µL. The present thermal cycling profile was elaborated from the original publication: Stage 1: 15 min at 95°C; Stage 2: 2 cycles of 15 s at 94°C, 15 s at 49°C; and Stage 3: 32 cycles of 15 s at 94°C, 10 s at 62°C, 15 s at 73°C with signal acquisition, 10 s at 84°C, 15 s at 87°C with signal acquisition; Stage 4: 1 cycle of 0.05 s at 65°C with signal acquisition. SYBR Green I, in QuantiFast, bind to double stranded DNA to produce a fluorescence signal. The 73°C reads provided the Ct values for the amplification of the telomere template (in early cycles when the albumin signal is still at baseline). The 87°C reads provided the Ct values for the amplification of the albumin template (at this temperature the telomere template is fully melted). The 65°C signal acquisition in Stage 4 was used to identify wells with primer-dimer formation for rerun. On each 384-well plate, DNA from participants was examined in quadruplicates together with triplicates of an internal control and a calibrator consisting of genomic DNA from the cell lines NTERA-2 and K562, respectively (purchased from DMSZ, Braunschweig, Germany). To determine the absolute telomere length (absTL) in basepairs of our calibrator, we measured its T/S ratio relative to the reference DNA sample used in Dr. Cawthon's 2009 methods [37], and then calculated the calibrator's absTL as [(T/S)×(absTL of the reference DNA)]. The absTL of this reference DNA is provided by the slope of the linear regression line in Figure 5 of ref. [38], since that slope equals the change in mean Terminal Restriction Fragment length (in basepairs) by Southern blot, per unit change in T/S, and the reference DNA, by definition, has T/S = 1.0 unit. The absTL of the calibrator derived in this way was 5,290 basepairs, which was comparable to previous reports for this cell line of 5,360 and 6,500 basepairs [39], [40]. Ct values are the raw results from a quantitative PCR analysis. Ct is short for cycle threshold, which is the number of PCR cycles needed to produce enough amplicons to raise the SYBR Green I fluorescence signal above a computer set threshold of the background fluorescence. T/S ratios were calculated as the cycle threshold value of the telomere amplicon divided by the cycle threshold value of the albumin amplicon [38].

#### Assay efficiency

DNA from each participant was measured in four different wells, resulting in four Ct values for the telomere signal and four Ct values for the albumin signal. A mean of the four telomere Ct values and the four albumin Ct values were calculated, and wells producing measurements outside mean Ct values ±2 SD were excluded, and new mean Ct values for telomere and albumin were calculated after exclusion of these outlier wells. Participants with less than two valid telomere and albumin Ct values were run again. For each participant we calculated a mean Ct value for the telomere assay (Cttel X) and for the albumin assay (Ctalb X). Similarly for the calibrator, we calculated means for Cttel and Ctalb across all plates (Cttel K562_All and Ctalb K562_All) and for each individual plate (Cttel K562_plate_X and Ctalb K562_plate_X). We calculated normalization factors for both assays on every plate. For the telomere assay, the normalization factor was: factortel_plate_X = (Cttel K562_All/Cttel K562_plate_X). Next, we calibrated Cttel and Ctalb of internal controls and participant samples. For the telomere assay, the calibration was: Cttel X _calibrated = Cttel X * factortel_plate_X. We then used the ΔΔCt calculation to derive the unknown relative telomere length of a participant sample (X): ΔCtX = Ctalb X_calibrated – Cttel X_calibrated. ΔCt K562 = Ctalb K562 – Cttel K562 and ΔΔCt = ΔCtX – ΔCt K562. The absolute telomere length of sample X was then calculated as = 2∧ΔΔCt * 5290 basepairs. Failed samples were measured a second round and a third if they failed again. Therefore, valid measurements of relative telomere lengths were available for more than 99.9% of participants.

#### Assay precision

The coefficient of variation was measured with the use of triplicates in each plate of DNA from the cell line NTERA-2, where ΔCt and the absolute telomere length were calculated exactly as for participant samples. Means and standard deviations were calculated for Cttel NTERA-2 and absolute telomere length values. The inter-assay coefficient of variation (CV) was determined using the calculation: CV = (standard deviation/mean)*100%. Coefficients of variation for the internal control were 1.8% for Cttel NTERA-2 at mean value of 18.0 and 9.3% for absolute telomere length in basepairs at the mean level of 2,577 basepairs. Intra-assay participant CV was 0 to 5.1% for Cttel and 0 to 3.6% for Ctalb.

Participants gave blood twice for DNA extraction with a roughly 10 years interval, and at both extractions we used the Qiagen blood kit. A median of 9.3 years passed between the first and second examination (SD = 0.43 years). We calculated 10 year change in telomere length using the formula: ((2001–03 telomere length – 1991–95 telomere length)/((2001–03 examination date – 1991–94 examination date)/365.25days)))*10 years. Thus, for each individual person we used the exact time difference between the two measurements and then converted it to a 10 year change.

### Statistical analyses

We used the statistical software package STATA version 11.1 for analysis (StataCorp, College Station, Texas). All statistical tests were two-sided. Information on age, gender, and year of birth was complete, while information on other covariates was more than 99% complete. For multivariable analyses, missing continuous covariates were imputed based on age and gender, whereas missing categorical values were assigned to a missing category.

As quantitative PCR for relative telomere length measurement is sensitive to DNA quality, type of DNA extraction, time between sample collection and processing, and as there is some degree of variation among runs, we already made sure that differences in DNA storage and isolation would not produce a false overall change (reduction or elongation alike) in relative telomere length. We did this by performing a linear regression between the 1991–94 examination relative telomere length and age. The coefficients from this analysis were used to calibrate the association between the 2001–03 relative telomere length measurement and age, and thereby ensuring that the association between relative telomere length and age remained, while keeping the measured variance from the 2001–03 measurement. As this calibration reduced the likelihood that differences in DNA isolation and storage would produce a false overall change (reduction or elongation alike) in relative telomere length, it also means that we cannot examine the average change in relative telomere length in the whole population over 10 years, but we can accurately look at relative changes in telomere length within each individual.

In the cross-sectional analysis, participants were categorized according to examination specific quartiles of telomere length for trend tests. Individuals in the 1^st^ quartile had the longest telomere length and individuals in the 4^th^ quartile had the shortest telomere length.

Absolute 10 years change in telomere length in basepairs was calculated as ((telomere length in basepairs at the 2001–2003 examination – telomere length in basepairs at the 1991–1994 examination)/(years between examinations))*10. Percent 10 years change in telomere length was calculated as ((telomere length in basepairs at the 2001–2003 examination - telomere length in basepairs at the 1991–1994 examination)/(telomere length in basepairs at the 1991–1994 examination))*100%. We used linear regression to associate leukocyte change with age at baseline (years), baseline tobacco consumption (g/day), tobacco consumption between examinations (g/day), baseline body weight (kg), body weight change (%), baseline alcohol intake (units of 12 g alcohol/day), alcohol intake between examinations (units of 12 g alcohol/day), baseline physical activity (hours/week), and physical activity between examinations (hours/week). Baseline was the 1991–1994 examination.

For prospective analysis we tested the proportional hazards assumption visually by plotting -ln(-ln(survival)) versus ln(analysis time): no violations were observed. Study entry for prospective analysis was defined as the day of blood sampling at the 2001–2003 examination, except for the analysis of mortality as a function of telomere length after each examination separately (Figure 4), where follow-up began at the day of examination. Participants were categorized according to quartiles of telomere length change. Individuals in the 1^st^ quartile gained the most and individuals in the 4^th^ quartile lost the most telomere basepairs between examinations. Follow-up began at the day of blood sampling at the 2001–2003 examination, and ended at death, diagnosis of endpoint of interest, emigration (n = 39), or June 7^th^ 2011 (for death endpoint), December 31^st^ 2009 (for cancer endpoint), and May 10^th^ 2011 (for chronic obstructive pulmonary disease, diabetes mellitus type II, ischemic cerebrovascular disease, and ischemic heart disease endpoints). Participants diagnosed with an endpoint of interest before the day of blood sampling at the 2001–2003 examination were excluded from analysis. For this reason numbers of participants among endpoints vary slightly. Multivariable adjustment included the following covariates obtained at the 2001–2003 examination: age (years), gender (male/female), current smoking (no/yes), daily tobacco consumption (g), body mass index (kg/m^2^), heavy alcohol intake (no/yes), and physical inactivity (no/yes).

## Supporting Information

Figure S1 Distribution (full curves) and medians (dotted lines) of telomere lengths among 4,576 participants of the 1991–94 (red) and the 2001–03 examination (blue).
(PDF)

Figure S2 Regression to the mean of telomere length. Median and interquartile ranges of telomere length for each quartile of 4,576 participants of the 1991–94 examination (left) and the same individuals, while maintaining the 1991–94 quartilation at the 2001–03 examination. Regression dilution ratio is 1069/2155 = 0.50.
(PDF)

Table S1 Characteristics of participants in the general population, the Copenhagen City Heart Study.
(DOC)

Table S2 Independent predictors of change in telomere length from the 1991–1994 to the 2001–2013 examinations in participants with telomere gain or loss.
(DOC)

Table S3 Mortality and morbidity by quartiles of telomere length change in participants with telomere gain or loss.
(DOC)
