# Supplementary figures and images for: Telomere Shortening Unrelated to Smoking, Body Weight, Physical Activity, and Alcohol Intake: 4,576 General Population Individuals with Repeat Measurements 10 Years Apart
Source: PLoS Genet. 2014 Mar 13;10(3):e1004191. doi: 10.1371/journal.pgen.1004191 (PMC3953026; doi:10.1371/journal.pgen.1004191)

Number of  
participants

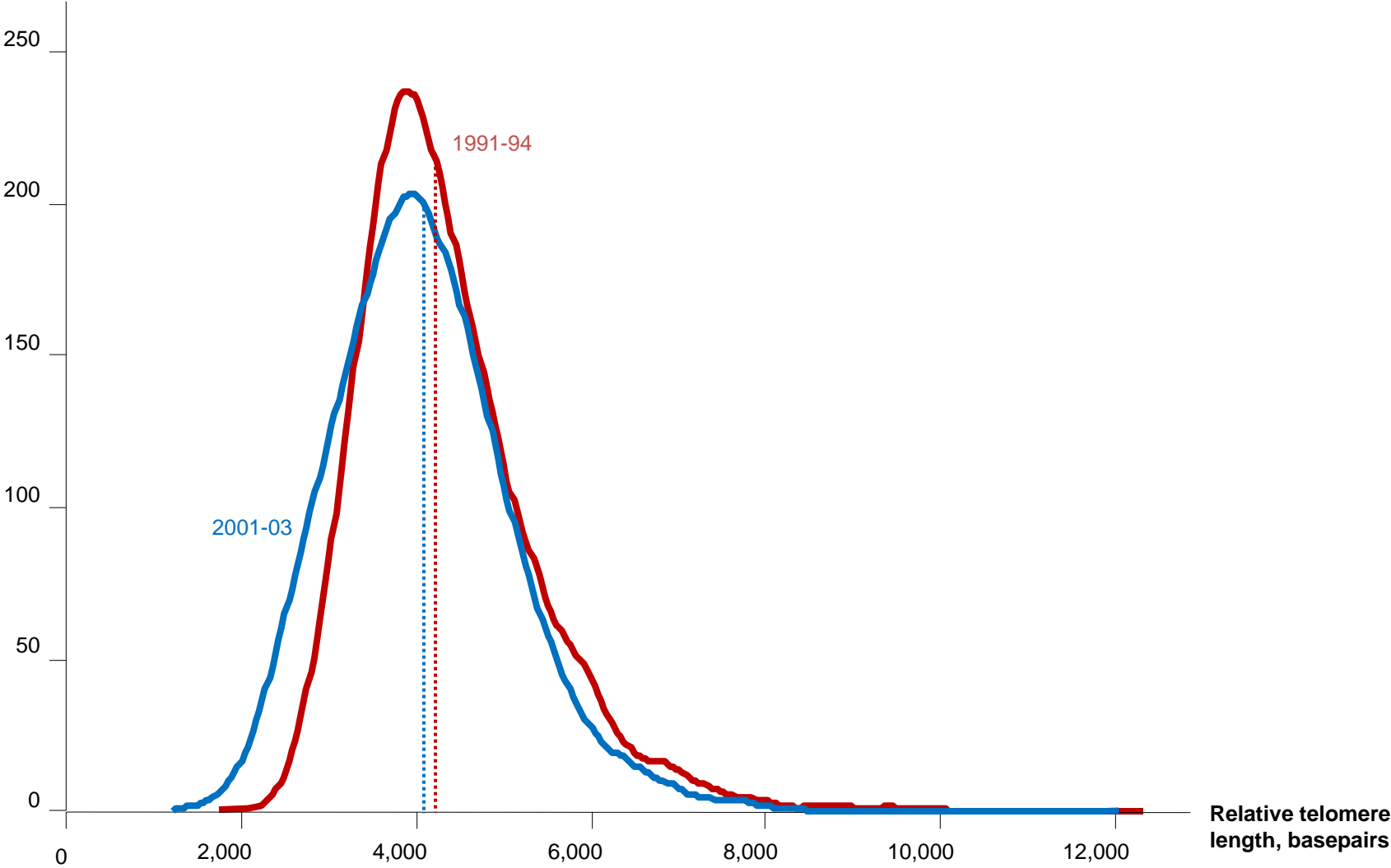

Supplement: Figure S1 — Distribution (full curves) and medians (dotted lines) of telomere lengths among 4,576 participants of the 1991–94 (red) and the 2001–03 examination (blue). (PDF) [file pgen.1004191.s001.pdf]

Relative telomere  
length, basepairs

1991-94  
Quartiles

1991-94  
Quartiles

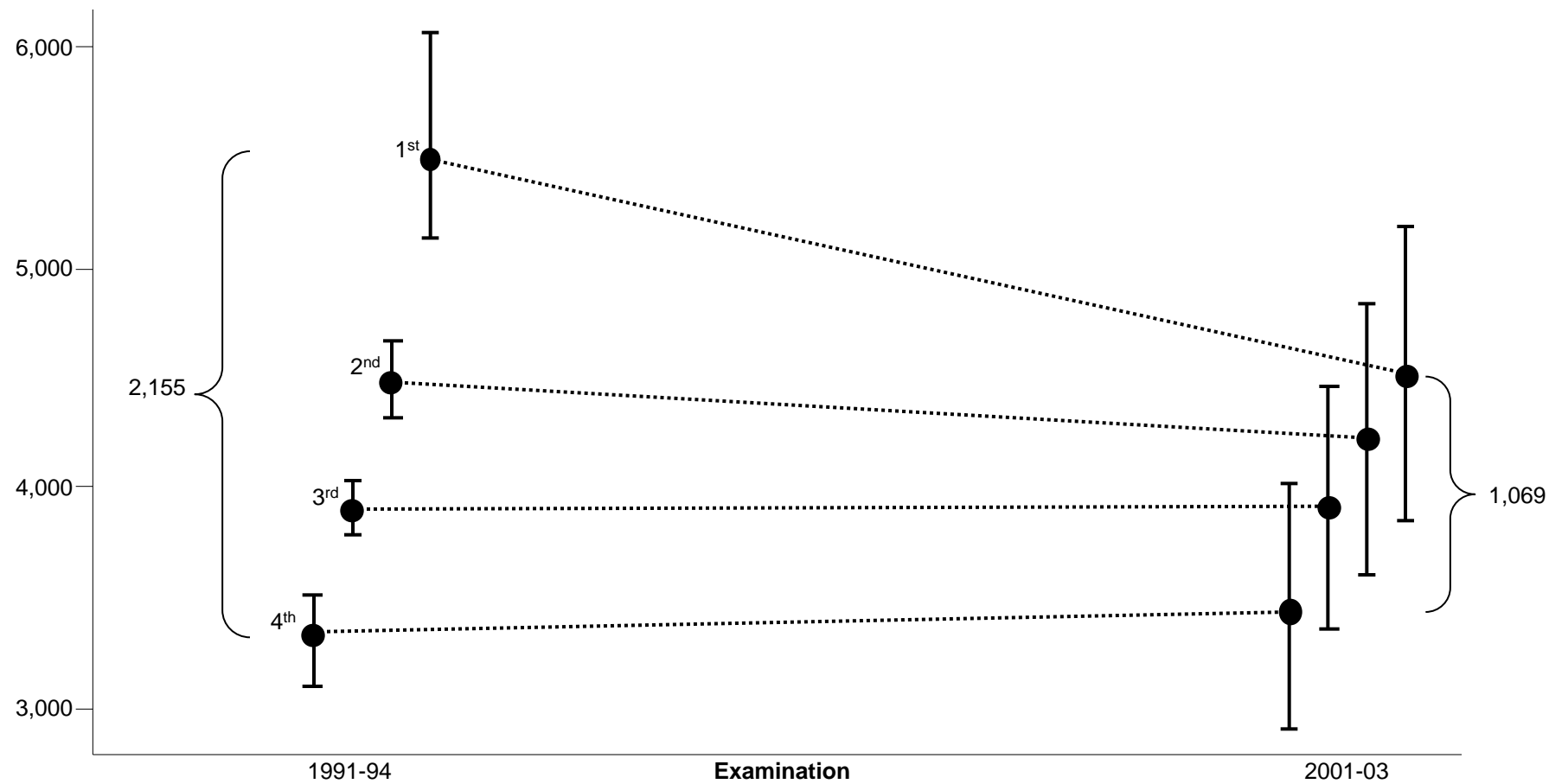

Supplement: Figure S2 — Regression to the mean of telomere length. Median and interquartile ranges of telomere length for each quartile of 4,576 participants of the 1991–94 examination (left) and the same individuals, while maintaining the 1991–94 quartilation at the 2001–03 examination. Regression dilution ratio is 1069/2155 = 0.50. (PDF) [file pgen.1004191.s002.pdf]
