# Supplementary material for: Telomere Shortening Unrelated to Smoking, Body Weight, Physical Activity, and Alcohol Intake: 4,576 General Population Individuals with Repeat Measurements 10 Years Apart
Source: PLoS Genet. 2014 Mar 13;10(3):e1004191. doi: 10.1371/journal.pgen.1004191 (PMC3953026; doi:10.1371/journal.pgen.1004191)
Supplement: Table S1 — Characteristics of participants in the general population, the Copenhagen City Heart Study. (DOC) [file pgen.1004191.s003.doc]

| | Table S1. Characteristics of participants in the general population, the Copenhagen City Heart Study. | | | | | | | --- | --- | --- | --- | --- | --- | |  | | 10 year change in telomere length | |  | | |  | |  | | |  | Shorter | | Longer | | *P* for trend | | No. of participants | 2,564 | | 2,012 | |  | | Men (%) | 1,114 (43) | | 829 (41) | | 0.13 | |  |  | |  | |  | | 1991-1994 examination |  | |  | |  | | Telomere length, basepairs | 12,132 to 2,361 | | 6,984 to 1,544 | | 6x10^-107^ | | Relative telomere length, T/S ratio | 0.83 to 0.69 | | 0.83 to 0.70 | | 1x10^-13^ | | Age, years | 55 (43 to 65) | | 54 (43 to 64) | | ? | | Current smoking, n (%) | 1104 (43) | | 873 (43) | | 0.98 | | Daily tobacco consumption, g * | 15 (10 to 20) | | 15 (10 to 20) | | 0.55 | | Body mass index, kg/m^2^ | 25 (22 to 27) | | 25 (22 to 27) | | 0.69 | | Physical inactivity, n (%)** | 451 (18) | | 351 (16) | | 0.20 | | Heavy alcohol intake, n (%)*** | 1,116 (44) | | 881 (44) | | 0.08 | |  |  | |  | |  | | 2001-2003 examination |  | |  | |  | | Telomere length, basepairs | 7,405 to 1,419 | | 12,088 to 2,435 | | 8x10^-264^ | | Relative telomere length, T/S ratio | 0.84 to 0.74 | | 0.84 to 0.73 | | 1x10^-94^ | | Age, years | 64 (31 to 95) | | 64 (32 to 93) | | 0.19 | | Current smoking, n(%) | 833 (32) | | 666 (33) | | 0.53 | | Daily tobacco consumption, g * | 15 (10 to 20) | | 15 (10 to 20) | | 0.12 | | Body mass index, kg/m^2^ | 26 (23 to 28) | | 26 (23 to 28) | | 0.23 | | Physical inactivity, n (%)** | 225 (9) | | 171 (9) | | 0.21 | | Heavy alcohol intake, n (%)*** | 1,156 (45) | | 913 (45) | | 0.98 | |
| --- | --- | --- | --- | --- | --- | --- | --- | --- | --- | --- | --- | --- | --- | --- | --- | --- | --- | --- | --- | --- | --- | --- | --- | --- | --- | --- | --- | --- | --- | --- | --- | --- | --- | --- | --- | --- | --- | --- | --- | --- | --- | --- | --- | --- | --- | --- | --- | --- | --- | --- | --- | --- | --- | --- | --- | --- | --- | --- | --- | --- | --- | --- | --- | --- | --- | --- | --- | --- | --- | --- | --- | --- | --- | --- | --- | --- | --- | --- | --- | --- | --- | --- | --- | --- | --- | --- | --- | --- | --- | --- | --- | --- | --- | --- | --- | --- | --- | --- | --- | --- | --- | --- | --- | --- | --- | --- | --- | --- | --- | --- | --- | --- | --- | --- | --- | --- | --- | --- | --- | --- | --- | --- | --- | --- | --- | --- | --- | --- | --- | --- | --- | --- | --- | --- | --- | --- | --- | --- | --- | --- | --- | --- | --- | --- | --- | --- | --- | --- | --- | --- | --- | --- | --- | --- |

Continuous variables are shown as medians (interquartile ranges) and categorical variables are shown as numbers (%).

* Smokers only.

** Less than four hour’s weekly physical activity. Covariates were more than 99% complete.

*** Weekly alcohol intake above 87.5 g in women and above 175 g in men. Only measured values are shown.
