# Supplementary material for: Telomere Shortening Unrelated to Smoking, Body Weight, Physical Activity, and Alcohol Intake: 4,576 General Population Individuals with Repeat Measurements 10 Years Apart
Source: PLoS Genet. 2014 Mar 13;10(3):e1004191. doi: 10.1371/journal.pgen.1004191 (PMC3953026; doi:10.1371/journal.pgen.1004191)
Supplement: Table S2 — Independent predictors of change in telomere length from the 1991–1994 to the 2001–2013 examinations in participants with telomere gain or loss. (DOC) [file pgen.1004191.s004.doc]

| Table S2 | | | | | | | | | | | | | | | | | | | | | | | | | | | | | | | | | | | | | | |
| --- | --- | --- | --- | --- | --- | --- | --- | --- | --- | --- | --- | --- | --- | --- | --- | --- | --- | --- | --- | --- | --- | --- | --- | --- | --- | --- | --- | --- | --- | --- | --- | --- | --- | --- | --- | --- | --- | --- |
|  |  | |  | | |  | | |  | | | | | | |  | | | |  | |  | | | | | | | |  | | | | | | |  | |
|  | | | | |  | | | 10 year change in telomere length, basepairs | | | | | | | | | | | |  | | | | | | 10 year change in telomere length, % | | | | | | | | | | | | |
|  | | Participants, n | | |  | | | β-coefficient | | | | | 95 %  confidence  interval | | | | *P*-value | | | | | | | |  | | β-coefficient | | | | | | 95 %  confidence  interval | | *P*-value | | | |
| **Particpants who gained telomere length** | |  | |  | | | | | |  | |  | | | | |  | | | | | | |  | | | | |  | |  | | |  | | | | |
| **Univariable analysis** | |  | |  | | | | | |  | |  | | | | |  | | | | | | |  | | | | |  | |  | | |  | | | | |
| Baseline telomere length, basepairs or % | | 2,012 | |  | | | 0.01 | | | | | -0.03 to 0.06 | | | | | 0.54 | | | | | | |  | | | | | -0.004 | | -0.005 to -0.003 | | | | 2x10^-15^ | | | |
| Relative telomere length, T/S ratio | | 2,012 | |  | | | -69.0 | | | | | -153 to -298 | | | | | 0.54 | | | | | | |  | | | | | -22.4 | | -27.8 to -16.9 | | | | 2x10^-15^ | | | |
| Age at baseline, years | | 2,012 | |  | | | -0.29 | | | | | -2.69 to 2.11 | | | | | 0.81 | | | | | | |  | | | | | -0.01 | | -0.07 to 0.04 | | | | 0.66 | | | |
| Baseline daily tobacco consumption, g/day | | 2,012 | |  | | | 2.26 | | | | | -0.84 to 5.36 | | | | | 0.15 | | | | | | |  | | | | | 0.07 | | -0.01 to 0.14 | | | | 0.09 | | | |
| Tobacco consumption between examinations, g/day | | 2,012 | |  | | | 2.84 | | | | | -0.57 to 6.25 | | | | | 0.10 | | | | | | |  | | | | | 0.07 | | -0.01 to 0.16 | | | | 0.10 | | | |
| Baseline weight, kg | | 2,012 | |  | | | 1.53 | | | | | -0.64 to 3.70 | | | | | 0.17 | | | | | | |  | | | | | 0.05 | | -0.01 to 0.10 | | | | 0.10 | | | |
| Weight change, % | | 2,012 | |  | | | 3.52 | | | | | 0.10 to 6.95 | | | | | 0.04 | | | | | | |  | | | | | 0.05 | | -0.38 to 0.13 | | | | 0.28 | | | |
| Baseline alcohol intake, units */day | | 2,012 | |  | | | 1.36 | | | | | -1.38 to 4.10 | | | | | 0.35 | | | | | | |  | | | | | 0.05 | | -0.02 to 0.12 | | | | 0.14 | | | |
| Alcohol intake between examinations, units */day | | 2,012 | |  | | | 0.28 | | | | | -2.76 to 3.33 | | | | | 0.86 | | | | | | |  | | | | | 0.01 | | -0.06 to 0.09 | | | | 0.71 | | | |
| Baseline physicial activity, hours/week | | 2,012 | |  | | | 19.20 | | | | | -3.56 to 41.94 | | | | | 0.10 | | | | | | |  | | | | | 0.02 | | -0.55 to 0.56 | | | | 0.93 | | | |
| Physical activity between examination, hours/week | | 2,012 | |  | | | 30.44 | | | | | 4.97 to 55.91 | | | | | 0.02 | | | | | | |  | | | | | 0.10 | | -0.54 to 0.74 | | | | 0.76 | | | |
| **Participants who gained telomere length** | |  | |  | | |  | | | |  | | | |  | | | |  | | | |  | | | | | | | | | | | | | | | |
| **Multivariable analysis** | |  | |  | | |  | | | |  | | | |  | | | |  | | | |  | | | | | | | | | | | | | | | |
| Baseline telomere length, basepairs or % | | 2,012 | |  | | | -0.006 | | | | | | | -0.05 to 0.04 | | | | 0.79 | | |  | | | | | | | -0.004 | | | | -0.006 to -0.003 | | | 3x10^-16^ | | | |
| Relative telomere length, T/S ratio | | 2,012 | |  | | | -33.3 | | | | | | | -281 to 215 | | | | 0.79 | | |  | | | | | | | -22.8 | | | | -28.5 to -17.0 | | | 3x10^-16^ | | | |
| Age at baseline, years | | 2,012 | |  | | | -3.72 | | | | | | | -6.53 to -0.91 | | | | 0.01 | | |  | | | | | | | -0.10 | | | | -0.17 to -0.03 | | | 0.004 | | | |
| Baseline daily tobacco consumption, g/day | | 2,012 | |  | | | -2.44 | | | | | | | -11.39 to 6.50 | | | | 0.59 | | |  | | | | | | | -0.02 | | | | -0.24 to 0.20 | | | 0.84 | | | |
| Tobacco consumption between examinations, g/day | | 2,012 | |  | | | 4.80 | | | | | | | -5.05 to 14.65 | | | | 0.34 | | |  | | | | | | | 0.07 | | | | -0.18 to 0.31 | | | 0.59 | | | |
| Baseline weight, kg | | 2,012 | |  | | | 1.86 | | | | | | | -0.43 to 4.15 | | | | 0.11 | | |  | | | | | | | 0.04 | | | | -0.02 to 0.10 | | | 0.15 | | | |
| Weight change, % | | 2,012 | |  | | | 2.38 | | | | | | | -1.47 to 6.22 | | | | 0.23 | | |  | | | | | | | 0.06 | | | | -0.03 to 0.15 | | | 0.21 | | | |
| Baseline alcohol intake, units */day | | 2,012 | |  | | | 4.61 | | | | | | | -1.37 to 10.60 | | | | 0.13 | | |  | | | | | | | 0.16 | | | | 0.01 to 0.30 | | | 0.04 | | | |
| Alcohol intake between examinations, units */day | | 2,012 | |  | | | -5.26 | | | | | | | -11.74 to 1.22 | | | | 0.11 | | |  | | | | | | | -0.17 | | | | -0.32 to -0.01 | | | 0.04 | | | |
| Baseline physicial activity, hours/week | | 2,012 | |  | | | -9.52 | | | | | | | -33.84 to 14.81 | | | | 0.44 | | |  | | | | | | | -0.28 | | | | -0.88 to 0.32 | | | 0.36 | | | |
| Physical activity between examination, hours/week | | 2,012 | |  | | | 2.26 | | | | | | | -30.89 to 35.41 | | | | 0.89 | | |  | | | | | | | 0.004 | | | | -0.81 to 0.82 | | | 0.99 | | | |
| **Participants who lost telomere length** | |  | |  | | |  | | | | |  | | | | |  | | | | | | |  | | | | |  | |  | | |  | | | | |
| **Univariable analysis** | |  | |  | | |  | | | | |  | | | | |  | | | | | | |  | | | | |  | |  | | |  | | | | |
| Baseline telomere length, basepairs or % | | 2,564 | |  | | | -0.51 | | | | | -0.53 to -0.48 | | | | | 3x10^-291^ | | | | | | |  | | | | | -0.005 | | -0.005 to -0.004 | | | | 2x10^-108^ | | | |
| Relative telomere length, T/S ratio | | 2,564 | |  | | | -2,673 | | | | | -2,794 to -2,551 | | | | | 3x10^-291^ | | | | | | |  | | | | | -24.5 | | -26.8 to -22.3 | | | | 2x10^-108^ | | | |
| Age at baseline, years | | 2,564 | |  | | | 4.04 | | | | | 1.64 to 6.45 | | | | | 0.001 | | | | | | |  | | | | | 2x10^-4^ | | -0.04 to 0.04 | | | | 0.99 | | | |
| Baseline daily tobacco consumption, g/day | | 2,564 | |  | | | 1.31 | | | | | -2.13 to 4.76 | | | | | 0.46 | | | | | | |  | | | | | 0.02 | | -0.04 to 0.07 | | | | 0.63 | | | |
| Tobacco consumption between examinations, g/day | | 2,564 | |  | | | 1.40 | | | | | -2.40 to 5.21 | | | | | 0.47 | | | | | | |  | | | | | 0.01 | | -0.03 to 0.05 | | | | 0.63 | | | |
| Baseline weight, kg | | 2,564 | |  | | | 1.09 | | | | | -1.27 to 3.45 | | | | | 0.37 | | | | | | |  | | | | | 0.02 | | -0.04 TO -0.07 | | | | 0.57 | | | |
| Weight change, % | | 2,564 | |  | | | -3.61 | | | | | -7.37 to 0.16 | | | | | 0.06 | | | | | | |  | | | | | -0.04 | | -0.10 TO 0.02 | | | | 0.22 | | | |
| Baseline alcohol intake, units */day | | 2,564 | |  | | | -2.44 | | | | | -5.49 to 0.60 | | | | | 0.12 | | | | | | |  | | | | | -0.05 | | -0.10 to -0.004 | | | | 0.03 | | | |
| Alcohol intake between examinations, units */day | | 2,564 | |  | | | -2.55 | | | | | -5.99 to 0.90 | | | | | 0.15 | | | | | | |  | | | | | -0.04 | | -0.09 to 0.02 | | | | 0.17 | | | |
| Baseline physicial activity, hours/week | | 2,564 | |  | | | -27.44 | | | | | -51.88 to -3.01 | | | | | 0.03 | | | | | | |  | | | | | -0.15 | | -0.52 to 0.23 | | | | 0.45 | | | |
| Physical activity between examination, hours/week | | 2,564 | |  | | | -38.03 | | | | | -66.56 to -9.49 | | | | | 0.01 | | | | | | |  | | | | | -0.09 | | -0.53 to 0.35 | | | | 0.68 | | | |
| **Participants who lost telomere length** | |  | |  | | |  | | | |  | | | |  | | | |  | | | |  | | | | | | | | | | | | | | | |
| **Multivariable analysis** | |  | |  | | |  | | | |  | | | |  | | | |  | | | |  | | | | | | | | | | | | | | | |
| Baseline telomere length, basepairs or % | | 2,564 | |  | | | -0.51 | | | | | | | -0.53 to -0.49 | | | | 3x10^-292^ | | |  | | | | | | | -0.005 | | | | -0.005 to -0.004 | | | 2x10^-118^ | | | |
| Relative telomere length, T/S ratio | | 2,564 | |  | | | -2,693 | | | | | | | -2,819 to -2,568 | | | | 3x10^-292^ | | |  | | | | | | | -24.5 | | | | -26.8 to -22.1 | | | 2x10^-118^ | | | |
| Age at baseline, years | | 2,564 | |  | | | -8.25 | | | | | | | -10.52 to -5.98 | | | | 1x10^-12^ | | |  | | | | | | | -0.12 | | | | -0.16 to -0.08^3^ | | | 1x10^-8^ | | | |
| Baseline daily tobacco consumption, g/day | | 2,564 | |  | | | -1.72 | | | | | | | -9.61 to 6.12 | | | | 0.62 | | |  | | | | | | | 0.02 | | | | -0.13 to 0.16 | | | 0.84 | | | |
| Tobacco consumption between examinations, g/day | | 2,564 | |  | | | 2.51 | | | | | | | -6.27 to 11.29 | | | | 0.56 | | |  | | | | | | | -0.001 | | | | -+.17 to 0.16 | | | 0.99 | | | |
| Baseline weight, kg | | 2,564 | |  | | | -0.67 | | | | | | | -2.52 to 1.20 | | | | 0.49 | | |  | | | | | | | -3x10^-3^ | | | | -0.04 to 0.03 | | | 0.83 | | | |
| Weight change, % | | 2,564 | |  | | | -0.35 | | | | | | | -3.5 to 2.8 | | | | 0.83 | | |  | | | | | | | -0.03 | | | | -0.09 to 0.03 | | | 0.26 | | | |
| Baseline alcohol intake, units */day | | 2,564 | |  | | | -6.02 | | | | | | | -11.60 to -0.48 | | | | 0.04 | | |  | | | | | | | -0.13 | | | | -0.24 to -0.03 | | | 0.01 | | | |
| Alcohol intake between examinations, units */day | | 2,564 | |  | | | 3.31 | | | | | | | -28.30 to 9.45 | | | | 0.29 | | |  | | | | | | | 0.09 | | | | -0.03 to 0.20 | | | 0.13 | | | |
| Baseline physicial activity, hours/week | | 2,564 | |  | | | -8.00 | | | | | | | -28.10 to 12.07 | | | | 0.43 | | |  | | | | | | | -0.06 | | | | -0.43 to 0.32 | | | 0.76 | | | |
| Physical activity between examination, hours/week | | 2,564 | |  | | | -1.18 | | | | | | | -28.68 to 26.33 | | | | 0.93 | | |  | | | | | | | -0.05 | | | | -0.56 to 0.47 | | | 0.85 | | | |
| * One unit alcohol equals 12 g. The rate of change in leukocyte telomere length is negative; therefore, negative regression coefficients denote faster telomere length loss. Baseline values were obtained at the 1991-94 examination. | | | | | | | | | | | | | | | | | | | | | | | | | | | | | | | | | | | |  | |  |
