# Supplementary material for: Telomere Shortening Unrelated to Smoking, Body Weight, Physical Activity, and Alcohol Intake: 4,576 General Population Individuals with Repeat Measurements 10 Years Apart
Source: PLoS Genet. 2014 Mar 13;10(3):e1004191. doi: 10.1371/journal.pgen.1004191 (PMC3953026; doi:10.1371/journal.pgen.1004191)
Supplement: Table S3 — Mortality and morbidity by quartiles of telomere length change in participants with telomere gain or loss. (DOC) [file pgen.1004191.s005.doc]

| Table S3 | | | | | | | | | | | | | | | | | | | | | | | | | | | |  | | | | |  | | | | | |  |  |
| --- | --- | --- | --- | --- | --- | --- | --- | --- | --- | --- | --- | --- | --- | --- | --- | --- | --- | --- | --- | --- | --- | --- | --- | --- | --- | --- | --- | --- | --- | --- | --- | --- | --- | --- | --- | --- | --- | --- | --- | --- |
|  | |  |  | |  |  | | | | |  | |  | | | | | |  | | | | | | | |  |  | | | | | | | | | | |  |  |
|  | | 10 year change in telomere length,  Basepairs T/S ratio | | | | |  |  | | |  | | | Hazard ratio (95% confidence inteval) | | | | | | | | | | | | | | | | |  | | |  | | | | |  |  |
|  | |  |  |  |  |  |  | Participants, n | | | | Events, n | | | | | | Unadjusted | | | Multivariably adjusted | | | | | | |  | | | *P* for trend | | | | | | | |  |  |
|  | |  |  | |  |  | |  |  | | | | | | | |  | | | | | | |  | |  | | | | | | | | | | | | |  |  |
| **Participants who gained telomere length lengthrelative telomere length telomere lengthrelative telomere length** | | | | | |  | |  |  | | | | | | | |  | | | | | | |  | |  | | | | | | | | | | | | |  |  |
| All cause mortality | |  |  | |  |  | |  |  | | | | | | | |  | | | | | | |  | |  | | | | | | | | | | | | |  |  |
| 1^st^ quartile | | 1 to 265 | | | | 2x10^-4^ to 0.050 | | 503 | | | 104 | | | | | 1.00 | | | | | | 1.00 | | | | | | | | 0.43 | | | | | | | | | |  |
| 2^nd^ quartile | | 266 to 562 | | | | 0.051 to 0.106 | | 503 | | | 106 | | | | | 1.09(0.83 to 1.43) | | | | | | 1.12(0.85 to 1.48) | | | | | | | | | |  | | | | | | | | |
| 3^rd^ quartile | | 563 to 1,016 | | | | 0.107 to 0.192 | | 503 | | | 85 | | | | | 0.83(0.62 to 1.11) | | | | | | 0.90(0.67 to 1.20) | | | | | | | | | |  | | | | | | | | |
| 4^th^ quartile | | 1,017 to 7,278 | | | | 0.193 to 1.38 | | 503 | | | 76 | | | | | 0.92(0.68 to 1.24) | | | | | | 0.95(0.70 to 1.28) | | | | | | | | | |  | | | | | | | | |
|  | |  |  | | |  | |  | | |  | | | | |  | | | | | |  | | | | | | | | | |  | | | | | | | | |
| Cancer | |  |  | | |  | |  | |  | | | |  | | | | | |  | | | | | | | |  | | |  | | | | | | | |  |  |
| 1^st^ quartile | | 1 to 265 | | | | 2x10^-4^ to 0.050 | | 470 | | | 45 | | | 1.00 | | | | | | | | | 1.00 | | | | | 0.92 | | | | | | | | | | |  |  |
| 2^nd^ quartile | | 266 to 562 | | | | 0.051 to 0.106 | | 469 | | | 57 | | | 1.31(0.89 to 1.94) | | | | | | | | | 1.30(0.88 to 1.93) | | | | |  | | | | | | | | | | |  |  |
| 3^rd^ quartile | | 563 to 1,016 | | | | 0.107 to 0.192 | | 464 | | | 41 | | | 0.93(0.61 to 1.42) | | | | | | | | | 0.94(0.62 to 1.44) | | | | |  | | | | | | | | | | |  |  |
| 4^th^ quartile | | 1,017 to 7,278 | | | | 0.193 to 1.38 | | 473 | | | 47 | | | 1.15(0.77 to 1.74) | | | | | | | | | 1.14(0.76 to 1.72) | | | | |  | | | | | | | | | | |  |  |
|  |  | |  | |  | |  | |  | | | | |  | | | | | | | | | |  | | |  | | | | | | | | | |  |  |  |  |
| Chronic obstructive pulmonary disease | |  | | | |  | |  | | |  | | |  | | | | | | | | |  | | | | | |  | | | | | | | | | |  |  |
| 1^st^ quartile | | 1 to 265 | | | | 2x10^-4^ to 0.050 | | 490 | | | 40 | | | 1.00 | | | | | | | | | 1.00 | | | | | | 0.16 | | | | | | | | | |  |  |
| 2^nd^ quartile | | 266 to 562 | | | | 0.051 to 0.106 | | 491 | | | 32 | | | 0.80(0.50 to 1.27) | | | | | | | | | 0.77(0.48 to 1.24) | | | | | |  | | | | | | | | | |  |  |
| 3^rd^ quartile | | 563 to 1,016 | | | | 0.107 to 0.192 | | 487 | | | 26 | | | 0.64(0.39 to 1.05) | | | | | | | | | 0.72(0.44 to 1.18) | | | | | |  | | | | | | | | | |  |  |
| 4^th^ quartile | | 1,017 to 7,278 | | | | 0.193 to 1.38 | | 491 | | | 26 | | | 0.70(0.42 to 1.14) | | | | | | | | | 0.71(0.43 to 1.18) | | | | | |  | | | | | | | | | |  |  |
|  | |  |  | | |  | |  | | |  | | |  | | | | | | | | |  | | | | | |  | | | | | | | | | |  |  |
| Diabetes Mellitus type II | | | | | |  | |  | | |  | | |  | | | | | | | | |  | | | | | |  | | | | | | | | | |  |  |
| 1^st^ quartile | | 1 to 265 | | | | 2x10^-4^ to 0.050 | | 488 | | | 24 | | | 1.00 | | | | | | | | | 1.00 | | | | | | 0.20 | | | | | | | | | |  |  |
| 2^nd^ quartile | | 266 to 562 | | | | 0.051 to 0.106 | | 489 | | | 25 | | | 1.07(0.61 to 1.88) | | | | | | | | | 1.13(0.64 to 1.99) | | | | | |  | | | | | | | | | |  |  |
| 3^rd^ quartile | | 563 to 1,016 | | | | 0.107 to 0.192 | | 487 | | | 21 | | | 0.88(0.49 to 1.58) | | | | | | | | | 0.95(0.53 to 1.71) | | | | | |  | | | | | | | | | |  |  |
| 4^th^ quartile | | 1,017 to 7,278 | | | | 0.193 to 1.38 | | 491 | | | 13 | | | 0.60(0.30 to 1.17) | | | | | | | | | 0.64(0.32 to 1.27) | | | | | |  | | | | | | | | | |  |  |
|  | |  |  | | |  | |  | | |  | | |  | | | | | | | | |  | | | | | |  | | | | | | | | | |  |  |
| Ischemic cerebrovascular disease | | | | | |  | |  | | |  | | |  | | | | | | | | |  | | | | | |  | | | | | | | | | |  |  |
| 1^st^ quartile | | 1 to 265 | | | | 2x10^-4^ to 0.050 | | 492 | | | 23 | | | 1.00 | | | | | | | | | 1.00 | | | | | | 0.95 | | | | | | | | | |  |  |
| 2^nd^ quartile | | 266 to 562 | | | | 0.051 to 0.106 | | 484 | | | 29 | | | 1.32(0.76 to 2.29) | | | | | | | | | 1.29(0.75 to 2.24) | | | | | |  | | | | | | | | | |  |  |
| 3^rd^ quartile | | 563 to 1,016 | | | | 0.107 to 0.192 | | 491 | | | 24 | | | 1.05(0.59 to 1.87) | | | | | | | | | 1.10(0.62 to 1.96) | | | | | |  | | | | | | | | | |  |  |
| 4^th^ quartile | | 1,017 to 7,278 | | | | 0.193 to 1.38 | | 494 | | | 20 | | | 1.01(0.55 to 1.84) | | | | | | | | | 1.03(0.56 to 1.88) | | | | | |  | | | | | | | | | |  |  |
|  | |  |  | | |  | |  | | |  | | |  | | | | | | | | |  | | | | | |  | | | | | | | | | |  |  |
| Ischemic heart disease | | | | | |  | |  | | |  | | |  | | | | | | | | |  | | | | | |  | | | | | | | | | |  |  |
| 1^st^ quartile | | 1 to 265 | | | | 2x10^-4^ to 0.050 | | 459 | | | 57 | | | 1.00 | | | | | | | | | 1.00 | | | | | | 0.87 | | | | | | | | | |  |  |
| 2^nd^ quartile | | 266 to 562 | | | | 0.051 to 0.106 | | 471 | | | 40 | | | 0.69(0.46 to 1.03) | | | | | | | | | 0.69(0.46 to 1.03) | | | | | |  | | | | | | | | | |  |  |
| 3^rd^ quartile | | 563 to 1,016 | | | | 0.107 to 0.192 | | 464 | | | 42 | | | 0.70(0.47 to 1.05) | | | | | | | | | 0.74(0.50 to 1.11) | | | | | |  | | | | | | | | | |  |  |
| 4^th^ quartile | | 1,017 to 7,278 | | | | 0.193 to 1.38 | |  | | |  | | |  | | | | | | | | |  | | | | | |  | | | | | | | | | |  |  |
|  | |  | | | |  | |  | | |  | | |  | | | | | | | | |  | | | | | |  | | | | | | | | | |  |  |
| **Participants who lost telomere length** | | | | | |  | |  | | |  | | |  | | | | | | | | |  | | | | | |  | | | | | | |  |  | |  |  |
| All cause mortality | |  | | | |  | |  | | |  | | |  | | | | | | | | |  | | | | | |  | | | | | |  | |  | |  |  |
| 1^st^ quartile | | 0 to -325 | | | | 0 to -0.060 | | 641 | | | 130 | | | 1.00 | | | | | | | | | 1.00 | | | | | | 0.11 | | | | | | | | | |  |  |
| 2^nd^ quartile | | -326 to -707 | | | | -0.061 to -0.133 | | 641 | | | 117 | | | 1.03(0.80 to 1.32) | | | | | | | | | 0.98(0.76 to 1.26) | | | | | |  | | | | | | | | | |  |  |
| 3^rd^ quartile | | -708 to -1,286 | | | | -0.134 to -0.243 | | 641 | | | 136 | | | 1.07(0.84 to 1.36) | | | | | | | | | 1.11(0.87 to 1.41) | | | | | |  | | | | | | | | | |  |  |
| 4^th^ quartile | | -1,287 to -8,406 | | | | -0.244 to -1.590 | | 641 | | | 122 | | | 1.13(0.88 to 1.45) | | | | | | | | | 1.19(0.93 to 1.52) | | | | | |  | | | | | | | | | |  |  |
|  | |  | | | |  | |  | | |  | | |  | | | | | | | | |  | | | | |  | | | | | | | | | | |  |  |
| Cancer | |  | | | |  | |  | | |  | | |  | | | | | | | | |  | | | | |  | | | | | | | | | | |  |  |
| 1^st^ quartile | | 0 to -325 | | | | 0 to -0.060 | | 588 | | | 48 | | | 1.00 | | | | | | | | | 1.00 | | | | | 0.12 | | | | | | | | | | |  |  |
| 2^nd^ quartile | | -326 to -707 | | | | -0.061 to -0.133 | | 591 | | | 53 | | | 1.13(0.77 to 1.67) | | | | | | | | | 1.08(0.73 to 1.59) | | | | |  | | | | | | | | | | |  |  |
| 3^rd^ quartile | | -708 to -1,286 | | | | -0.134 to -0.243 | | 586 | | | 72 | | | 1.50(1.04 to 2.17) | | | | | | | | | 1.55(1.07 to 2.23) | | | | |  | | | | | | | | | |  |  |  |
| 4^th^ quartile | | -1,287 to -8,406 | | | | -0.244 to -1.590 | | 588 | | | 52 | | | 1.18(0.80 to 1.75) | | | | | | | | | 1.21(0.82 to 1.80) | | | | |  | | | | | | | | | |  |  |  |
|  | |  | | | |  | |  | | |  | | |  | | | | | | | | |  | | | | |  | | | | | | | | | |  |  |  |
| Chronic obstructive pulmonary disease | |  | | | |  | |  | | |  | | |  | | | | | | | | |  | | | | |  | | | | | | | | | |  |  |  |
| 1^st^ quartile | | 0 to -325 | | | | 0 to -0.060 | | 624 | | | 49 | | | 1.00 | | | | | | | | | 1.00 | | | | | 0.79 | | | | | | | | | |  |  |  |
| 2^nd^ quartile | | -326 to -707 | | | | -0.061 to -0.133 | | 625 | | | 44 | | | 0.96(0.64 to 1.44) | | | | | | | | | 0.88(0.58 to 1.33) | | | | |  | | | | | | | | | |  |  |  |
| 3^rd^ quartile | | -708 to -1,286 | | | | -0.134 to -0.243 | | 619 | | | 40 | | | 0.82(0.54 to 1.24) | | | | | | | | | 0.92(0.60 to 1.40) | | | | |  | | | | | | | | | |  |  |  |
| 4^th^ quartile | | -1,287 to -8,406 | | | | -0.244 to -1.590 | | 614 | | | 37 | | | 0.85(0.55 to 1.30) | | | | | | | | | 0.93(0.61 to 1.44) | | | | |  | | | | | | | | | |  |  |  |
|  | |  | | | |  | |  | | |  | | |  | | | | | | | | |  | | | | |  | | | | | | | | | |  |  |  |
| Diabetes Mellitus type II | |  | | | |  | |  | | |  | | |  | | | | | | | | |  | | | | |  | | | | | | | | | |  |  |  |
| 1^st^ quartile | | 0 to -325 | | | | 0 to -0.060 | | 621 | | | 23 | | | 1.00 | | | | | | | | | 1.00 | | | | | 0.27 | | | | | | | | | |  |  |  |
| 2^nd^ quartile | | -326 to -707 | | | | -0.061 to -0.133 | | 617 | | | 30 | | | 1.36(0.79 to 2.35) | | | | | | | | | 1.37(0.79 to 2.36) | | | | |  | | | | | | | | | |  |  |  |
| 3^rd^ quartile | | -708 to -1,286 | | | | -0.134 to -0.243 | | 627 | | | 33 | | | 1.42(0.83 to 2.41) | | | | | | | | | 1.46(0.85 to 2.50) | | | | |  | | | | | | | | | |  |  |  |
| 4^th^ quartile | | -1,287 to -8,406 | | | | -0.244 to -1.590 | | 613 | | | 26 | | | 1.22(0.69 to 2.13) | | | | | | | | | 1.36(0.77 to 2.40) | | | | |  | | | | | | | | | |  |  |  |
|  | |  | | | |  | |  | | |  | | |  | | | | | | | | |  | | | | |  | | | | | | | | | |  |  |  |
| Ischemic cerebrovascular disease | |  | | | |  | |  | | |  | | |  | | | | | | | | |  | | | | |  | | | | | | | | | |  |  |  |
| 1^st^ quartile | | 0 to -325 | | | | 0 to -0.060 | | 622 | | | 27 | | | 1.00 | | | | | | | | | 1.00 | | | | | 0.76 | | | | | | | | | |  |  |  |
| 2^nd^ quartile | | -326 to -707 | | | | -0.061 to -0.133 | | 614 | | | 31 | | | 1.30(0.77 to 2.18) | | | | | | | | | 1.30(0.77 to 2.19) | | | | |  | | | | | | | | | |  |  |  |
| 3^rd^ quartile | | -708 to -1,286 | | | | -0.134 to -0.243 | | 625 | | | 31 | | | 1.20(0.71 to 2.01) | | | | | | | | | 1.23(0.73 to 2.06) | | | | |  | | | | | | | | | |  |  |  |
| 4^th^ quartile | | -1,287 to -8,406 | | | | -0.244 to -1.590 | | 625 | | | 25 | | | 1.06(0.62 to 1.84) | | | | | | | | | 1.10(0.64 to 1.91) | | | | |  | | | | | | | | | |  |  |  |
|  | |  | | | |  | |  | | |  | | |  | | | | | | | | |  | | | | |  | | | | | | | | | |  |  |  |
| Ischemic heart disease | |  | | | |  | |  | | |  | | |  | | | | | | | | |  | | | | |  | | | | | | | | | |  |  |  |
| 1^st^ quartile | | 0 to -325 | | | | 0 to -0.060 | | 590 | | | 49 | | | 1.00 | | | | | | | | | 1.00 | | | | | 0.16 | | | | | | | | | |  |  |  |
| 2^nd^ quartile | | -326 to -707 | | | | -0.061 to -0.133 | | 587 | | | 63 | | | 1.39(0.96 to 2.03) | | | | | | | | | 1.33(0.92 to 1.94) | | | | |  | | | | | | | | | |  |  |  |
| 3^rd^ quartile | | -708 to -1,286 | | | | -0.134 to -0.243 | | 581 | | | 80 | | | 1.71(1.20 to 2.44) | | | | | | | | | 1.74(1.22 to 2.49) | | | | |  | | | | | | | | | |  |  |  |
| 4^th^ quartile | | -1,287 to -8,406 | | | | -0.244 to -1.590 | | 595 | | | 53 | | | 1.15(0.78 to 1.70) | | | | | | | | | 1.20(0.81 to 1.77) | | | | |  | | | | | | | | | |  |  |  |
